# Library of Ohio College of Dental Surgery

**Published:** 1869-11

**Authors:** 


### LIBRARY OF OHIO COLLEGE OF DENTAL SURGERY.

This library is being collected, arranged and put in proper form for the use of the Institution. It is desirable that this library shall embrace the entire Dental literature, so far as it can be procured, all text, standard and journalistic works, together with the principal standard works of medicine and surgery.

Such a collection will necessarily involve considerable expense, and we would here suggest that contributions of books or journals, dental or medical, will be thankfully received and acknowledged. The book or books in every instance, unless objected to by the doner, will bear his name.

We trust that at least some contribution will be received from every friend of the institution, and especially from every stockholder. Send on your contributions, gentlemen, don't be afraid of overdoing the matter. Quite a large number of books are already pledged. A fine library and reading room is fitted up in the college, where we trust very soon the student and practitioner will have an opportunity to consult any work pertaining to dental science or practice. T.

